# Effects of upper extremity blood flow restriction training on muscle strength and hypertrophy: a systematic review and meta-analysis

**DOI:** 10.3389/fphys.2024.1488305

**Published:** 2025-01-06

**Authors:** Jiapeng Jing, Qinzhi Zheng, Hongfei Dong, Yan Wang, Peiji Wang, Di Fan, Zhuo Xu

**Affiliations:** ^1^ Department of Rehabilitation, China-Japan Union Hospital of Jilin University, Changchun, China; ^2^ Rehabilitation Therapeutics, School of Nursing, Jilin University, Changchun, China

**Keywords:** blood flow restriction training, upper extremity muscle, muscle strength, muscle hypertrophy, adult

## Abstract

**Background:**

Low load resistance training with blood flow restriction (LL-BFRT) has been shown to improve muscle strength and hypertrophic function. The effect of LL-BFRT on lower extremity muscle improvement has been widely discussed. However, no studies have discussed the effect of this training method on the upper extremity muscles until now. This systematic review and meta-analysis focused on the use of LL-BFRT in the upper extremity muscles.

**Methods:**

The relevant literature was searched in four major databases including Pubmed, Web of science, the Cochrane Library and Embase from 10 June 2024. The Cochrane Collaboration’s tool and GRADE methodology were used to assess the risk of bias and quality in included studies.

**Results:**

The meta-analysis included a total of 11 articles with 220 participants. LL-BFRT and high load resistance training (HLRT) produced similar effects in improving upper extremity muscle strength (low certainty evidence, SMD: −0.35; 95%CI: −0.73 to 0.03; *p*: 0.07; I^2^: 2%) and hypertrophy (moderate certainty evidence, SMD: −0.36; 95%CI: −0.73 to 0.01; *p*: 0.05; I^2^: 0%). Compared with low load resistance training (LLRT), LL-BFRT showed greater advantages in improving upper extremity muscle strength (low certainty evidence, SMD: 0.67; 95%CI: 0.33 to 1.01; *p*: 0.0001; I^2^: 0%) and hypertrophy (low certainty evidence, SMD: 0.37; 95%CI: 0.06 to 0.67; *p*: 0.02; I^2^: 0%).

**Conclusion:**

In general, LL-BFRT can be used as an alternative training method for HLRT to improve upper extremity muscle strength and hypertrophy. Our study shows that the effect of LL-BFRT on upper extremity muscle is limited by age and region. It is necessary to formulate reasonable exercise programs according to the characteristics of different demographic groups.

**Systematic Review Registration:**

https://www.crd.york.ac.uk/PROSPERO/, identifier CRD42024555514.

## 1 Introduction

Muscle weakness that leads to reduced muscle strength and hypertrophy is commonly seen in musculoskeletal diseases. Muscle atrophy can be caused by prolonged immobilization or treatment of musculoskeletal diseases (Thomas et al., 2016). Studies have shown that muscle strength is an important predictor of cardiometabolic risk. A meta-analysis of 20,000 participants showed that increased muscle strength was associated with a reduced risk of death in adults (García-Hermoso et al., 2018). At the same time, decreased muscle strength is a major risk factor for osteoarthritis, the most common musculoskeletal disease, leading to a decline in patients’ daily quality of life (Petterson et al., 2008; Papalia et al., 2014). Therefore, improving muscle strength and atrophy that play a key role in protecting human health are essential in the rehabilitation of clinical musculoskeletal diseases.

According to recent reviews (Labata-Lezaun et al., 2020; Grgic et al., 2020), resistance training should be considered the primary treatment for improving muscle strength, mass, and hypertrophy. The American College of Sports Medicine recommends for resistance training load that improving muscle strength and hypertrophy should require at least 60%–70% of one maximum repetition (1RM) and 70%–85% of 1RM (American College of Sports Medicine position stand, 2009). However, high load resistance training (HLRT) is difficult to implement in the elderly or specific pathologies such as pain, muscle weakness and functional limitations (Kumar et al., 2009).

In recent years, low load resistance training with blood flow restriction (LL-BFRT) has been paid more and more attention (de Lemos Muller et al., 2024). LL-BFRT was first proposed by Dr. Yoshiaki Sato in Japan in the late 1970s, also known as “KAATSU training” in Japan (Chang et al., 2023). This training method is mainly used to apply external pressure to the limb during low load resistance training (20%–30% 1RM) with the help of special compression devices to mechanically restrict arteries and veins. The current researches suggest that LL-BFRT may produce similar effects to HLRT in terms of muscle strength and hypertrophy, and is superior to low load resistance training (LLRT) (Labata-Lezaun et al., 2022; Pavlou et al., 2023).

The effectiveness of LL-BFRT for increased muscle strength and mass has been proven, but its specific mechanism of action is still under discussion (Vopat et al., 2020). The mechanism proposed so far is mainly based on the synergistic effect of metabolic stress and mechanical stress. Under the combined action of these factors, the muscle is in the environment of ischemia, hypoxia and oxidative stress. Such an environment usually causes a buildup of lactic acid and reactive oxygen species, an increase in muscle synthesis-related hormones such as growth hormone, mobilization of type II fibers and ultimately an increase in muscle strength and hypertrophy (Pearson and Hussain, 2015).

In clinical practice, a large number of randomized controlled trials (RCT) have demonstrated the role of LL-BFRT in anterior cruciate ligament injury (Erickson et al., 2019), osteoarthritis (Hu et al., 2023; Sørensen et al., 2023) and patellofemoral pain (Liu and Wu, 2023; Kong W. et al., 2023). Several past systematic reviews have also explored the effects of LL-BFRT on muscle strength and hypertrophy in the lower extremity (Xiaolin et al., 2023; Wang et al., 2023), but few studies have focused on the upper extremity. Therefore, it is necessary to review the effects of LL-BFRT in upper extremity muscles based on recent research results.

The aim of this systematic review and meta-analysis was to systematically review the application of LL-BFRT to upper extremity muscles and to compared the effects of LL-BFRT with HLRT and LLRT on upper extremity muscle strength and hypertrophy. We also considered age and region as secondary factors.

## 2 Methods

### 2.1 Search strategy

This systematic review and meta-analysis adhered to the guidelines provide in the Preferred Reporting Items for Systematic Review and Meta-Analysis (PRISMA) (Page et al., 2021) and conducted with the recommendations of the Cochrane Handbook (Higgins et al., 2023). This study protocol was registered in PROSPERO with the number: CRD42024555514. Two researchers independently cross-checked eligible studies in four databases: Pubmed, Web of Science, the Cochrane Library, and Embase from the establishment of the database to 10 June 2024. The combination of Mesh terms and text related to two sections included in blood flow restriction training and upper extremity was used for study retrieval. To ensure that at least one search term is included in the results, all synonyms were connected with the operator “OR,” and both parts were connected with the operator “And.” The searcher strategy was detailed in Supplementary Table S1.

### 2.2 Inclusion and exclusion criteria

The inclusion criteria were based on PICOS (Population-Intervention-Comparison-Outcome- Study design) strategy. 1) Population: the study participants must at least 18 years old with or without any disease; 2) Intervention: the experimental group was treated with LL-BFRT, and the control group was treated with LLRT (<30%1RM) or HLRT (>60%1RM); 3) Comparison: the study design allowed comparison of difference between LL-BFRT group and LLRT group or HLRT group; 4) Outcomes: pre- and post-training measures of biceps brachii and/or triceps brachii strength and/or size; 5) Study design: randomized controlled trials written in English.

We excluded clinical trials based the following criteria: 1) experiment performed with animals as subjects; 2) non-original studies (experiment protocols, meeting abstract, review, etc.); 3) non-randomized controlled trial; 4) the experimental group and the control group were compared with the same intervention object on both sides of the upper limbs; 5) literature with full text or valid indicators was not available; 6) articles not published in English.

### 2.3 Study selection and data extraction

The relevant studies from the four databases were imported into Endnote. The articles were independently screened by two researchers according to the inclusion and exclusion criteria, and any differences were ruled by the third researcher. Extract data including the first author, publication year, study region, population characteristics, exercise and intervention characteristics and the main conclusion of the study.

### 2.4 Quality assessment

Each included RCTs quality was evaluated by two researchers using The Cochrane Collaboration’s tool (Higgins et al., 2011). The evaluation tool was consisted of seven items assessing the random sequence generation, the allocation concealment, the blinding of participants and personnel, the blinding of outcome assessment, the incomplete outcome data, the selective reporting and other bias. The quality of each item was rated as low risk, high risk, or unclear and was indicated by three difference colors. If there was a disagreement between the two researchers on the results, the third researcher with judging ability was decided.

### 2.5 Certainty of evidence

The certainty of the evidence was assessed independently by two reviewers using the Grading of Recommendations, Assessment, Development and Evaluation (GRADE) method (Guyatt et al., 2008). Since only RCT studies were included in this review five downgrading factors were considered: risk of bias, inconsistency, indirectness, imprecision and publication bias. The strength of the evidence was rated as “high,” “moderate,” “low,” or “very low.” Any differences were resolved by the third researcher.

### 2.6 Statistical analysis

Statistical analysis was performed using RevMan (Review Manager Version 5.4, The Cochrane Collaboration, 2020). Because of the obvious differences in the methods taken to measure muscle strength and hypertrophy across the studies and between each outcome, we used standardized mean difference (SMD) and 95% confidence intervals (95% CI) to summarized the effect size. Using the Mean and standard deviation (SD) to calculated the overall effect size, we define the Mean_change_ formula: Mean_change_ = Mean_post_ - Mean_pre_, the SD_change_ formula: SD_change_ = root square (SD_pre_^2^ + SD_post_^2^) – 2 * correlation * SD_pre_ * SD_post_. The correlation was set to 0.5 (Zhu et al., 2023). The random effects model was adopted for analysis, observing the measured variability and heterogeneity between the studies according to I^2^. The pooled effect size (ES) was calculated for each comparison and the alpha level was set to *p* < 0.05. Data were expressed as mean ± standard deviation. Results were considered to have low heterogeneity (I^2^ less than 25%), moderate (I^2^ between 25% and 75%) and high (I^2^ more than 75%).

This review compared the increase in muscle strength and hypertrophy between LL-BRF group and HLRT group or LLRT groups. We also performed subgroup analyses of age and region of the subjects to understand the effects of different conditions on muscle strength and hypertrophy. A meta-analysis was performed only when the data for analyzed variables were represented in at least 2 studies/comparisons.

In order to determine whether any RCTs biased the results of the combination, we performed a sensitivity analysis by removing each RCT. A study was considered to results the bias when the estimate after the elimination of a research surpassed the 95% CI for the combined effect. An Egger’s test using Stata version 16 (Stata Corp LP, College Station, TX, United States) was used to check for potential publication bias.

## 3 Result

### 3.1 Study selection

**FIGURE 1:**
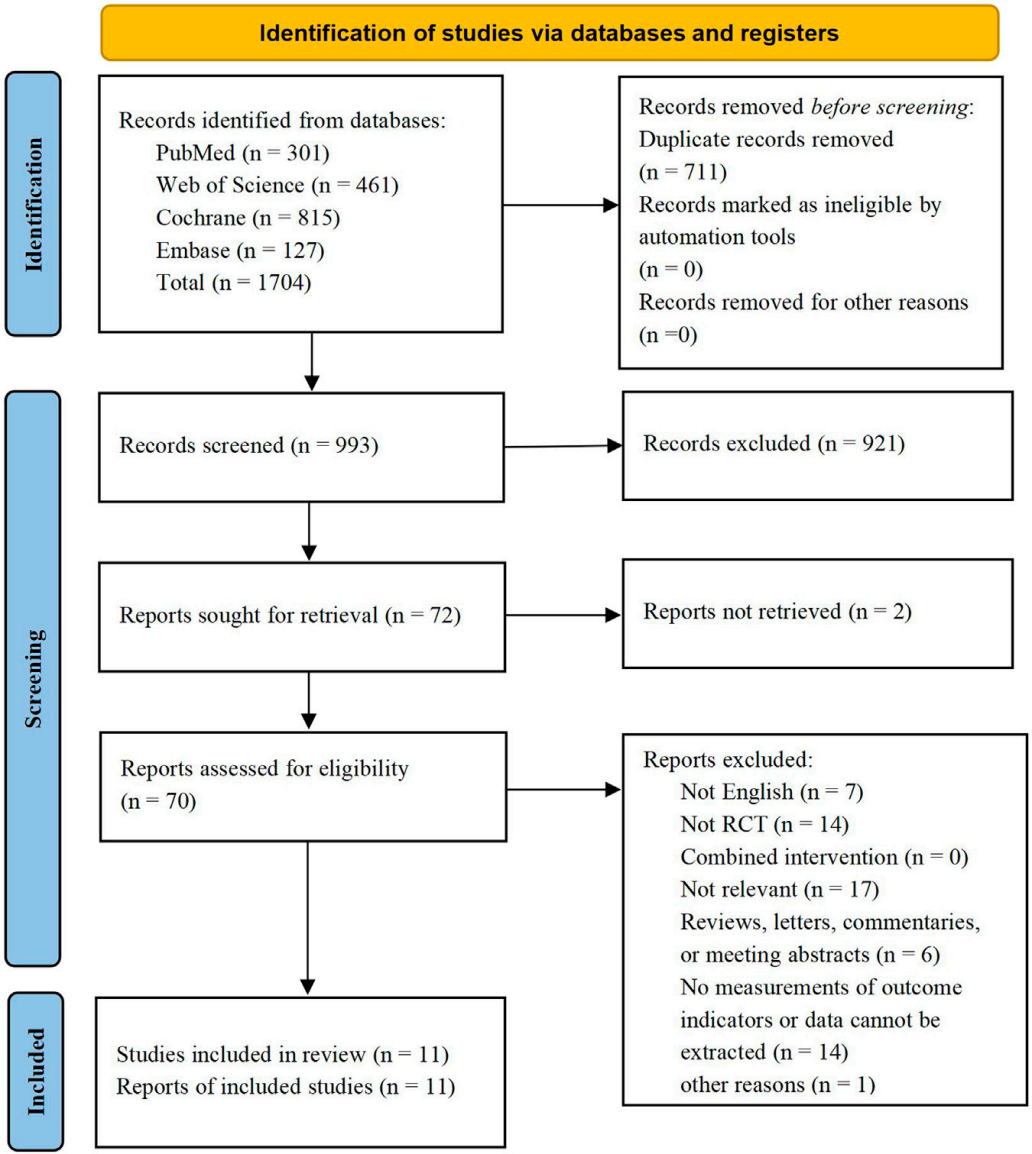
PRISMA flow diagram.

A total of 1,704 relevant articles were retrieved from four databases, and 711 duplicate records were subsequently excluded. A careful review of the titles and abstracts led to the exclusion of 921 studies and 2 studies were excluded due to they could not be retrieved. After checking for eligibility of these articles based on our inclusion and exclusion criteria, 59 articles were excluded: not English (n = 7); not RCT (n = 14); Not relevant (n = 17); reviews, letters, commentaries, or meeting abstracts (n = 6); no measurements of outcome indicators or data cannot be extracted (n = 14) and other reasons (n = 1). By comparing the main information of the included literatures, it was found that the first author and publication time of the two literatures were exactly identical (Yasuda et al., 2011a; Yasuda et al., 2011b). After further analysis the objects, groups, interventions and measures of the two literatures, we concluded that the two literatures came from the same experiment, therefore one of them was excluded (Yasuda et al., 2011a). Finally, 11 RCTs were included for data extraction. The studies screening process was shown in Figure 1.

### 3.2 Study characteristics and participants

A total of 11 RCTs (Yasuda et al., 2011b; Alves et al., 2021; Lowery et al., 2014; Brandner et al., 2019; Hill et al., 2020; Yasuda et al., 2015a; Yasuda et al., 2015b; Su et al., 2023; Linero and Choi, 2021; Wells et al., 2019; Kara et al., 2024) published between 2014-2024 were included, 6 from Asia, 3 from North America, both from South America and Australia were 1. We included a total of 220 participants, of whom 97 were male, 109 were female and 14 were of unknown gender. Of these studies, 6 studies discussed the differences between LL-BRF and HLRT (4 of them found that there was no significant between-group difference, and 2 concluded HLRT tends to result in better increase), 7 studies discussed the differences between LL-BFRT and LLRT (2 found that there were similar increases in LL-BFRT and LLRT, and 5 concluded LL-BFRT tends to result in greater increase). Among all included articles, 3 articles were only recruited males, 4 articles were only enrolled females, 3 articles did not distinguish gender and 1 article failed to provide gender of subjects; the participants of 9 studies were healthy and 2 studies were unhealthy; 2 RCTs involved older adults (over 60 years old) and 6 involved young adults (under 30 years old). In addition, details of each research background were shown in Table 1.

**TABLE 1:** Main information extracted from included studies.

Study	Region	Subjects	Protocol	Pressure measurement	N	Gender (M/F)	Duration; Frequency	Sets × repetitions	Exercise	Conclusion
Thiago Cândido Alves et al., 2021	Brazil	AIDS Patients (30–60 years)	LL-BFRT 100% AOP 30% 1RM HLRT 80% 1RM	Subjects were supine with cuffs placed proximally on the arms, and radial blood pressure measured using a vascular Doppler probe.	7 7	NA	12 wk; 3 days/wk	3 × maximum	EE EF	No significant between-group difference.
Christopher R. Brandner et al., 2019	Australia	Young Adults (23 ± 3 years)	LL-BFRT 60% LOP 20% 1RM LLRT 20% 1RM HLRT 70% 1RM	Subjects were seated with cuffs placed proximally on the arms, and LOP measured using a LOP sensor kit.	11 10 11	22/10	8 wk; 3 days/wk	LL-BRR/LLRT 3 × 15 HLRT 3 × (8-10)	EE BP	HLRT tends to result in best strength increase. Similar increases in LL-BFRT group and LLRT group.
Christian Linero et al., 2021	South Korea	Postmenopausal Females (50–60 years)	LL-BFRT 152 ± 6 mmHg 30% 1RM LLRT 30% 1RM HLRT 60%–80% 1RM	NA	7 6 7	0/20	12 wk; 3 days/wk	LL-BRR/LLRT 3 × 20 HLRT 3 × 10	EE EF	Best muscle strength gains for HLRT. LL-BFRT tends to result in greater strength increase than LLRT.
Tomohiro Yasuda et al., 2015 (1)	Japan	Older Adults (61–85 years)	LL-BFRT 196 ± 18 mmHg 20%–30% 1RM LLRT 20%–30% 1RM	NA	9 8	3/14	12 wk; 2 days/wk	1 × 30 + 3 × 15	EE EF	LL-BFRT tends to result in greater strength increase.
Ethan C. Hill et al., 2020	United States	Young Females (22 ± 2 years)	LL-BFRT 40% AOP 30% PT LLRT 30% PT	Subjects’ position was unspecified, cuffs were placed proximally on the arms, and brachial blood pressure was measured.	10 10	0/20	4 wk; 3 days/wk	1 × 30 + 3 × 15	EE EF	No significant between-group difference except for peak torque (greater in LL-BFRT).
Yanhong Su et al., 2024	China	Young Males (20–24 years)	LL-BFRT 140 mmHg 30% 1RM LLRT 30% 1RM	NA	9 9	18/0	12 wk; 5 days/wk	1 × 30 + 3 × 15	EE EF	LL-BFRT tends to result in greater strength increase.
Elizabeth WELLS et al., 2019	United States	Young Females (18–40 years)	LL-BFRT 60% LOP 30% 1RM HLRT 60% 1RM	NA	9 8	0/17	4 wk; 2 days/wk	LL-BFR 3 × 20 HLRT 3 × 10	EE	No significant between-group difference.
Ryan P. Lowery et al., 2014	United States	Young Males (23 ± 5 years)	LL-BFRT 60%–70% LOP 30% 1RM HLRT 60% 1RM	NA	10 10	20/0	4 wk; 2 days/wk	LL-BFR 3 × 30 HLRT 3 × 15	EE EF BP	No significant between-group difference.
Tomohiro Yasuda et al., 2011 (2)	Japan	Young Males (22–32 years)	LL-BFRT 100–160 mmHg 30% 1RM HLRT 75% 1RM	NA	10 10	20/0	6 wk; 3 days/wk	LL-BFR 1 × 30 + 3 × 15 HLRT 3 × 10	BP	No significant between-group difference.
Dilara Kara et al., 2024	Turkey	Young Adults with Rotator Cuff Tendinopathy (18–45 years)	LL-BFRT 50% LOP 30% 1RM LLRT 30% 1RM	Subjects were supine with cuffs placed proximally on the arms, and radial blood pressure measured using a vascular Doppler probe.	14 14	14/14	8 wk; 2 days/wk	1 × 30 + 3 × 15	EE	LL-BFRT tends to result in greater muscle hypertrophy increase.
Tomohiro Yasuda et al., 2015	Japan	Older Females (61–85 years)	LL-BFRT 202 ± 8 mmHg 20%–30% 1RM LLRT 20%–30% 1RM	NA	7 7	0/14	12 wk; 2 days/wk	1 × 30 + 3 × 15	EE EF	LL-BFRT tends to result in greater strength increase.

1RM, one maximum repetition; PT, peak torque; AOP, arterial occlusive pressure; LOP, limb occlusive pressure; EE, elbow extension; EF, elbow flexion; BP, bench press LL-BFRT, low load resistance with blood flow restriction training; HLRT, high load resistance training; LLRT, low load resistance training, M male, F female, wk week(s), NA, not available.

The numbers in brackets in the Study column indicate the order of publication by the same author in the same year.

### 3.3 Study intervention characteristics

In all articles included, the training load of the LL-BFRT and LLRT groups ranged from 20% to 30% 1RM, except for a reference with 30% PT, the training load of the HLRT group ranged from 60% to 80% 1RM. The training frequency of 5 articles was 2 days/wk, 5 articles was 3 days/wk, and 1 article was 5 days/wk. Training duration of 5 studies was 4 weeks, 1 study was 6 weeks, 2 studies was 8 weeks and 5 studies was 12 weeks. A total of 10 trials included elbow extension in the training program, 7 trials included elbow flexion in the training program, and 4 trials included bench press in the training program.

### 3.4 Outcome measure

Muscle strength including 1RM, maximum voluntary isometric contraction (MVIC) or peak torque (PT) was measured by isometric dynamometer or 1RM test in 9 articles, of which 8 articles measured elbow flexion and 5 articles measured elbow extension. Muscle hypertrophy including muscle cross-sectional area (CSA) or muscle thickness (MTH) was measured by MRI or ultrasound in 8 studies, of which 7 studies measured the biceps brachii and 4 studies measured the triceps brachii. Details of the outcome measure were shown in Supplementary Table S2.

### 3.5 Risk of bias assessment

**FIGURE 2:**
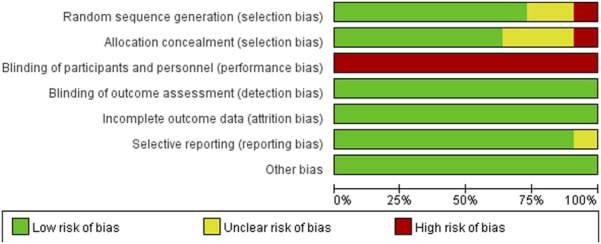
Risk of bias graph.

**FIGURE 3:**
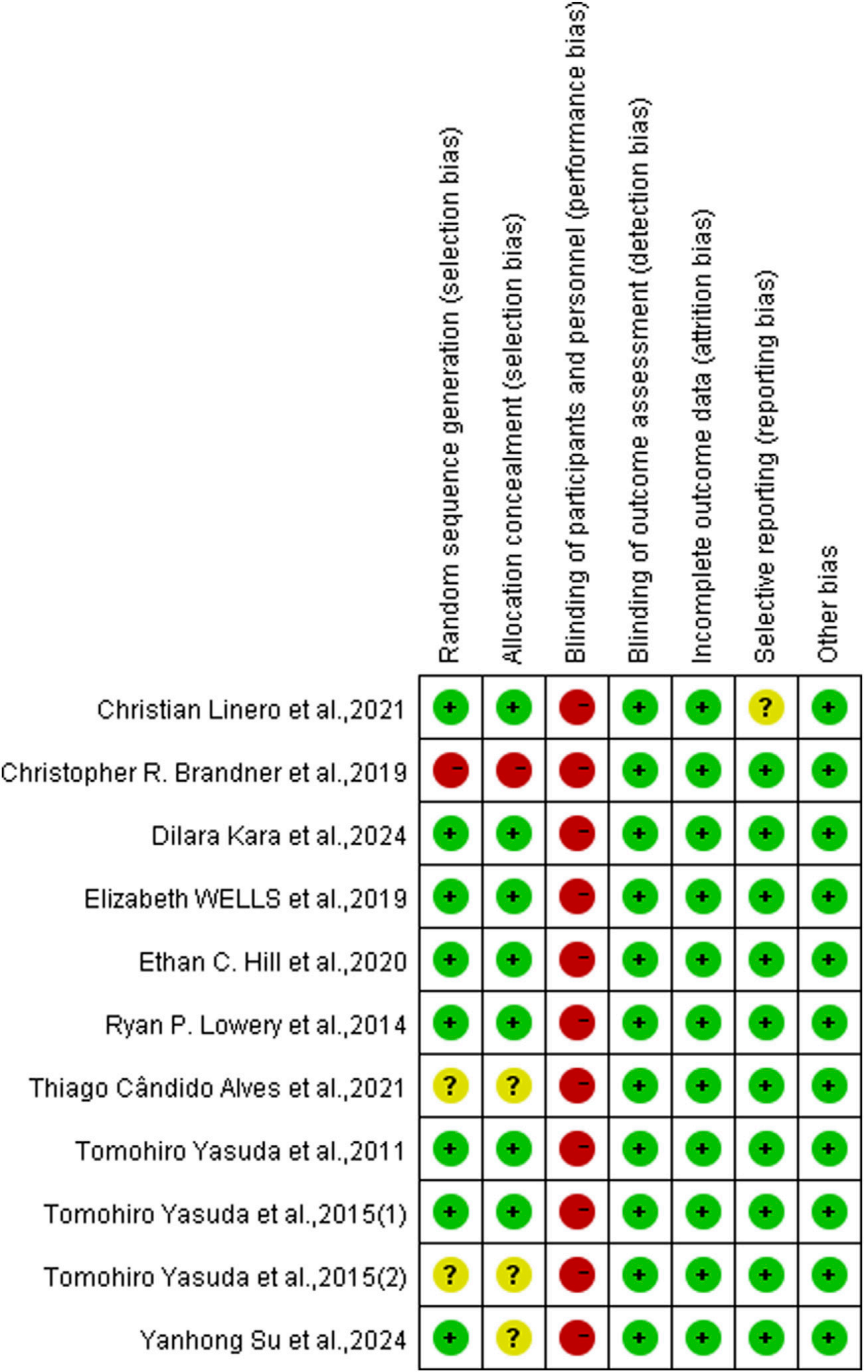
Risk of bias summary.

The included articles were assessed using the Cochrane risk of bias tool. Of the 11 studies included, 8 were assessed as having a low risk of selection bias, 2 were assessed as uncertain risk due to the randomization method was not explicitly reported, and 1 was assessed as high risk due to adjustments for participants. Three articles did not report allocation concealment as uncertain risk and 1 was assessed as high risk. Because the particularity of the intervention method included in the study, it was not feasible to blind researchers and subjects, so all of them were judged to be high risk of performance bias, but this did not affect the quality of the trial. Detection bias and attrition bias in all trials were performed to be low risk. One study was indicated a high risk of reporting bias, and no studies were found to have other bias. The studies risk of bias was shown in Figures 2, 3.

### 3.6 The effect of LL-BFRT compared with HIRT

**FIGURE 4:**
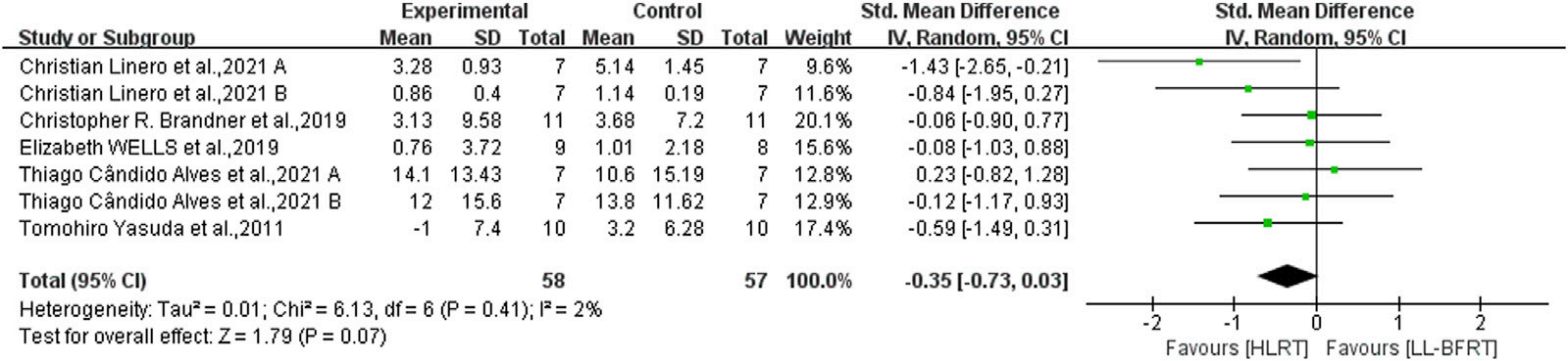
Forest plot depicts a comparison of upper extremity muscle strength between studies using LL-BFRT and studies using HLRT. Different letters represent different measurements of the same study.

**FIGURE 5:**
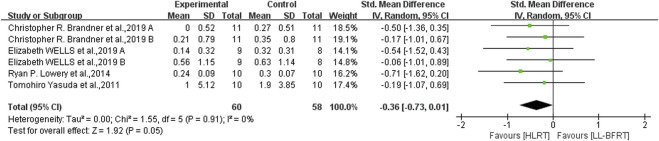
Forest plot depicts a comparison of upper extremity muscle hypertrophy between studies using LL-BFRT and studies using HLRT. Different letters represent different measurements of the same study.

Low certainty evidence from 5 articles (Yasuda et al., 2011b; Alves et al., 2021; Brandner et al., 2019; Linero and Choi, 2021; Wells et al., 2019) (7 comparisons; n = 115) suggested that LL-BFRT and HLRT were not statistically significant for increases in upper extremity muscle strength (SMD: −0.35; 95%CI: −0.73 to 0.03; *p*: 0.07; I^2^: 2%) (Table 2; Figure 4). Moderate certainty evidence from 4 articles (Yasuda et al., 2011b; Lowery et al., 2014; Brandner et al., 2019; Wells et al., 2019) (6 comparisons; n = 118) indicated that LL-BFRT and HLRT were not statistically significant for increases in upper extremity muscle hypertrophy (SMD: −0.36; 95%CI: −0.73 to 0.01; *p*: 0.05; I^2^: 0%) (Table 2; Figure 5). Leave-one-out sensitivity analyses showed that a single trial did not affect the significance of the overall changes in upper muscle strength and hypertrophy. For both upper muscle strength and hypertrophy, there was no significant heterogeneity by using funnel plots and Egger’s test (*p* < 0.05).

**TABLE 2:** Summery of evidence of the effects of LL-BFRT compared with LLRT or HLRT on muscle strength and hypertrophy.

Comparisons	Groups	N (comparisons)	SMD (95%CI)	I^2^ (%)	*p*-value	Quality of evidence (GRADE)
overall analysis						
LL-BFRT vs. HLRT in muscle strength	NA	7	−0.35 (−0.73, 0.03)	2	0.07	⊕〇〇〇 Low^a^ ^,^ ^d^
LL-BFRT vs. HLRT in muscle hypertrophy	NA	6	−0.36 (− 0.73, 0.01)	0	0.05	⊕⊕〇〇 Moderate^d^
LL-BFRT vs. LLRT in muscle strength	NA	9	0.67(0.33, 1.01)	0	0.0001	⊕〇〇〇 Low^a^ ^,^ ^d^
LL-BFRT vs. LLRT in muscle hypertrophy	NA	9	0.37 (0.06, 0.67)	0	0.02	⊕〇〇〇 Low^a^ ^,^ ^d^
subgroup analysis						
LL-BFRT vs. HLRT in muscle strength	Region					
	Asia	3	−0.87 (−1.48, -0.26)	0	0.0005	⊕〇〇〇 Low^a^ ^,^ ^d^
	America	3	0.00 (−0.58, 0.59)	0	0.99	⊕〇〇〇 Low^a^ ^,^ ^d^
LL-BFRT vs. HLRT in muscle hypertrophy	Region					
	America	3	−0.44 (−0.99, 0.10)	0	0.11	⊕⊕〇〇 Moderate^d^
	Australia	2	−0.33 (−0.93, 0.26)	0	0.27	⊕〇〇〇 Low^a^ ^,^ ^d^
LL-BFRT vs. LLRT in muscle strength	Age (year)					
	young (<30)	3	0.71 (0.17, 1.25)	0	0.0009	⊕〇〇〇 Low^a^ ^,^ ^d^
	older (>60)	4	0.48 (0.06, 1.91)	0	0.03	⊕〇〇〇 Low^a^ ^,^ ^d^
LL-BFRT vs. LLRT in muscle hypertrophy	Age (year)					
	young (<30)	4	0.01 (−0.42, 0.44)	0	0.96	⊕〇〇〇 Low^a^ ^,^ ^d^
	older (>60)	4	0.81 (0.28, 1.34)	0	0.003	⊕〇〇〇 Low^a^ ^,^ ^d^
	Region					
	Asia	4	0.81 (0.28, 1.34)	0	0.003	⊕〇〇〇 Low^a^ ^,^ ^d^
	America	3	0.23 (−0.26, 0.73)	0	0.36	⊕〇〇〇 Low^a^ ^,^ ^d^
	Australia	2	−0.04 (−0.64, 0.57)	0	0.90	⊕⊕〇〇 Moderate^a^

LL-BFRT, low load resistance with blood flow restriction training; HLRT, high load resistance training; LLRT, low load resistance training, NA not applicable; SMD, standardized mean difference.

^a^ Downgraded due to risk of bias.

^b^ Downgraded due to inconsistency.

^c^ Downgraded due to indirectness.

^d^ Downgraded due to imprecision.

^e^ Downgraded due to publication bias.

For muscle strength, subgroup meta-analysis showed that HLRT increased muscle strength more than LL-BFRT in the Asia subgroup (low certainty evidence; SMD: −0.87; 95%CI: −1.48 to −0.26; *p*: 0.0005; I^2^: 0%), but this difference was not found in the America subgroup (low certainty evidence; SMD: 0.00; 95%CI: −0.58 to 0.59; *p*: 0.99; I^2^:0%) (Table 2). In terms of muscle hypertrophy, we performed subgroup analyses by region, and the results showed that the outcomes for America (moderate certainty evidence; SMD: −0.44; 95%CI: −0.99 to 0.10; *p*: 0.11; I^2^: 0%) and Australia (low certainty evidence SMD: 0.33; 95%CI: −0.93 to 0.26; *p*: 0.27; I^2^: 0%) subgroups were consistent with the overall analysis (Table 2).

### 3.7 The effect of LL-BFRT compared with LLRT

**FIGURE 6:**
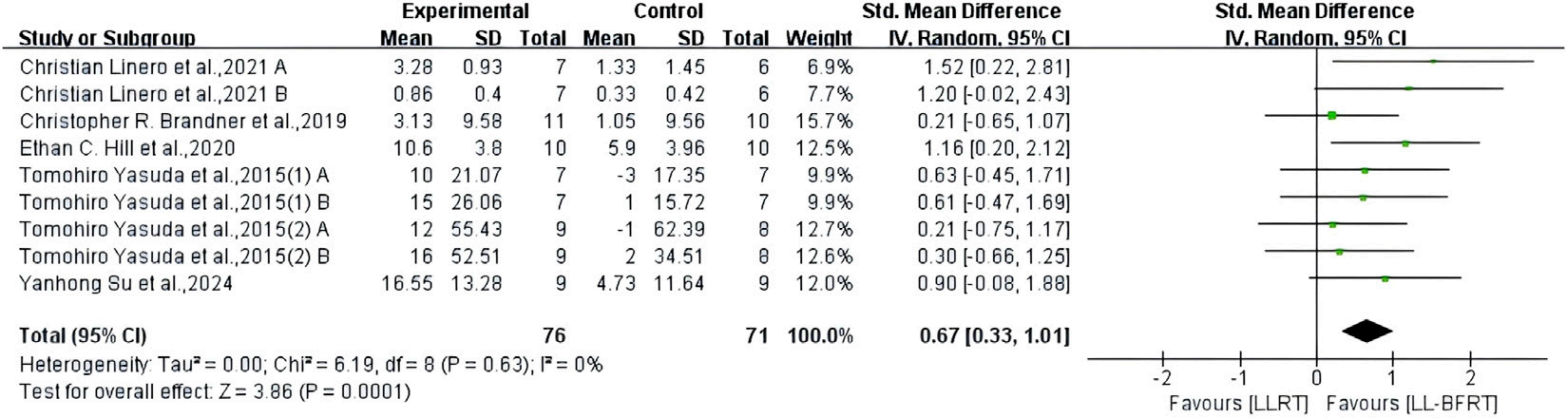
Forest plot depicts a comparison of upper extremity muscle strength between studies using LL-BFRT and studies using LLRT. Different letters represent different measurements of the same study. The numbers in parentheses represent the order of publication by the same author in the same year.

**FIGURE 7:**
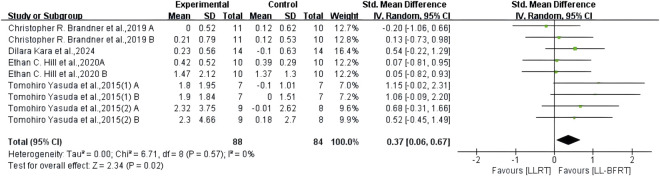
Forest plot depicts a comparison of upper extremity muscle hypertrophy between studies using LL-BFRT and studies using LLRT. Different letters represent different measurements of the same study. The numbers in parentheses represent the order of publication by the same author in the same year.

The results of meta-analysis including 6 RCTs (Brandner et al., 2019; Hill et al., 2020; Yasuda et al., 2015a; Yasuda et al., 2015b; Su et al., 2023; Linero and Choi, 2021) (9 compared; n = 147) showed low certainty evidence that LL-BFRT had a statistically significant increase in upper extremity muscle strength compared with LLRT (SMD: 0.67; 95%CI: 0.33 to 1.01; *p*: 0.0001; I^2^: 0%) (Table 2; Figure 6). There was low certainty evidence from 5 studies (Brandner et al., 2019; Hill et al., 2020; Yasuda et al., 2015a; Yasuda et al., 2015b; Kara et al., 2024) (9 compared; n = 172) that LL-BFRT performed a statistically significant increase in upper extremity muscle hypertrophy compared with LLRT (SMD: 0.37; 95%CI: 0.06 to 0.67; *p*: 0.02; I^2^: 0%) (Table 2; Figure 7). No single study had a significant impact on the overall SMD of both analyses. Funnel plot and egg’s test results showed no heterogeneity between studies in muscle strength and hypertrophy (*p* < 0.05).

We performed a subgroup analysis of muscle strength based on age factor and the results showed that LL-BFR increased muscle strength more significantly in both young (low certainty evidence; SMD: 0.71; 95%CI: 0.17 to 1.25; *p*: 0.0009; I^2^: 0%) and older adults (low certainty evidence; SMD: 0.48; 95%CI: 0.06 to 1.91; *p*: 0.03; I^2^: 0%) (Table 2). For muscle hypertrophy, two subgroup analyses were performed by age and region. Subgroup analysis of age showed that LL-BFRT increased muscle hypertrophy in older adults better than LLRT (low certainty evidence; SMD: 0.81; 95%CI: 0.28 to 1.34; *p*: 0.003; I^2^:0%), but this difference failed to be found in young adults (low certainty evidence; SMD: 0.01; 95%CI: −0.42 to 0.44; *p*: 0.96; I^2^: 0%) (Table 2). Subgroup analysis according to region suggested that LL-BFRT produced a significant increase in muscle hypertrophy in the Asia subgroup (low certainty evidence; SMD: 0.81; 95%CI: 0.28 to 1.34; *p*: 0.003; I^2^: 0%) not America (low certainty evidence; SMD: 0.23; 95%CI: −0.26 to 0.73; *p*: 0.36; I^2^:0%) and Australia subgroups (moderate certainty evidence; SMD: −0.04; 95%CI: 0.64 to 0.57; *p*: 0.90; I^2^: 0%) (Table 2) compared to LLRT.

## 4 Discussion

The purpose of this systematic review is to comprehensively analyze the effects of LL-BFRT on upper extremity muscle strength and hypertrophy compared with traditional HLRT and LLRT. The results of the review indicate that there is low quality evidence that LL-BFRT is similar in increasing upper extremity muscle strength HLRT and moderate quality evidence that LL-BFRT has no difference in improving upper muscle hypertrophy. Results on LL-BFRT *versus* LLRT provide low quality evidence that LL-BFRT led to better upper extremity muscle strength and hypertrophy effects compared to LLRT. We also performed a subgroup analysis of the overall effect by age and region. In terms of strength improvement, there was low quality evidence suggesting that LL-BFRT promoted lower strength gains compared to HLRT in the Asia subgroup. Subgroup analysis of hypertrophy confirmed that low-quality evidence showed that LL-BFRT did not induce more significant muscle hypertrophy in young adults (under 30 years) compared to LLRT, and the same results were found in the America (low quality evidence) and Australia (moderate quality evidence) subgroups. No inconsistency was found between the results of other subgroup analysis and the overall analysis. Unfortunately, there is not enough study to support a comprehensive subgroup analysis of each overall effect.

Our results show that HLRT has a significant advantage for upper extremity muscle strength and hypertrophy improvement, although there is no significant difference compared with LL-BFRT. This result is consistent with Kyriakos (Pavlou et al., 2023) previously published meta-analysis of four RCTs that found similar improvements in shoulder and back muscle strength between LL-BFRT and HLRT.

At present the effect of HLRT on improving muscle strength is beyond doubt (Izquierdo et al., 2021). Both training methods initially improve muscle strength through nerve recruitment to create efficient muscle contractions (Wells et al., 2019; Tennent et al., 2017). Some studies have observed the electromyography activity of the two training methods during exercise and found that the value of HLRT changes more significantly than that of LL-BFR (Cook et al., 2013; Manini and Clark, 2009). The increase in muscle strength caused by nerve recruitment has been found to be about 60% in young people and the effect may be even more pronounced in the elderly (Fabero-Garrido et al., 2022; Häkkinen et al., 2000). Compared to HLRT, the deficiency of LL-BFRT in muscle strength enhancement may be caused by insufficient nerve recruitment (Centner et al., 2019). At the same time, HLRT also creates greater mechanical stress on muscles and directly stimulates muscle fibers, especially Type II fibers, to improve strength more effectively (Cook et al., 2013; Kong J. et al., 2023). The American College of Sports Medicine recommendation to take a load of 70%–85% 1RM to improve muscle strength has become a widely accepted standard (American College of Sports Medicine position stand, 2009). However, HLRT may not work for everyone due to certain limitations (Franz et al., 2018). In contrast, LL-BFRT can be embraced by a broader group of people, such as the elderly, people with limited movement, and patients in recovery, providing a low-intensity, low-risk and effective training method that can improve muscle strength while reducing the risk of injury. There are also meta-analyses that have come to different conclusions than this study. Manoel E. Lixandra˜o et al. (Lixandrão et al., 2018) Compared 12 studies involving 460 participants and found that HLRT had a more significant and statistically significant increase in muscle strength compared with LL-BFRT. Luke Hughes et al. (Hughes et al., 2017) analyzed 5 studies and found that HLRT had a moderate effect on increased muscle strength compared to LL-BFRT. However, it is important to note that these reviews mainly focused on hip and knee muscle strength, so whether these conclusions can be applied to the upper extremity is uncertain, and more studies on upper extremity strength are needed to continue the discussion. The subgroup analysis of strength effects of LL-BFRT and HLRT showed that the two training methods had obvious regional effects on the improvement of muscle strength. We found a statistically significant increase in muscle strength in HLRT compared with LL-BFRT for Asians, but this effect was not observed in Americans. In previous reviews, few studies focused on the variable of region. But this is an aspect that we must pay attention to, the explosive power and endurance of muscles of different races are different (Leong et al., 2016). Therefore, the training method should not be consistent, only in this way can develop a more reasonable training program for different races.

Masses of studies have confirmed the effect of HLRT on muscle growth (Kong J. et al., 2023; Lichtenberg et al., 2019). Traditional resistance training believes that only the strong mechanical stress brought about by high intensity training can induce muscle growth (Pearson and Hussain, 2015). It has also been found that high intensity resistance training brings the greatest increase in muscle strength but muscle hypertrophy can be achieved under all kinds of resistance (Schoenfeld et al., 2017). It is obvious that the resistance during HLRT is greater and therefore the mechanical stress on the muscles is much greater than that of LL-BFRT. However, the related mechanisms that trigger the change of muscle morphology are not limited to mechanical stress, metabolic stress and muscle injury also have a certain contribution (Centner et al., 2019). One study found that LL-BFRT resulted in more lactic acid buildup compared to HLRT (Wells et al., 2019). The accumulation of metabolites represented by lactic acid can promote the growth and differentiation of muscle cells by increasing the metabolic pressure of muscle cells by activating the signal pathways related to muscle growth, such as MAPK pathway (Davids et al., 2023). In LL-BFRT, the muscle and its surrounding tissues exhibit different physiological characteristics from resistance training due to the factor of blood flow restriction. Pavlos Angelopoulos et al. suggest that the muscle hypertrophy effect produced by LL-BFRT has similar effects to HLRT (70% of maximum muscle strength) on muscle hypertrophy, strength, and cardiovascular response (Angelopoulos et al., 2023). It can be seen that both LL-BFRT and HLRT play an effective role in increasing upper extremity muscle hypertrophy. The results of subgroup analysis showed that the effects of LL-BFRT and HLRT on upper extremity muscle hypertrophy were not affected by region. However, due to the small number of researches, there is a probability of a small amount of sample deviation, so this conclusion needs to be carefully considered.

According to our analysis results, compared with LLRT, LL-BFRT has A significant and statistically significant improvement in upper extremity muscle strength and hypertrophy, which supports the previous analysis results of a previous review (Pavlou et al., 2023). Christoph Centner et al. (Davids et al., 2023) observed the application of LL-BFRT in the elderly population and found similar conclusions.

Compared with LLRT, LL-BFRT creates an anoxic environment for the muscles due to blood flow restriction, which increases the mechanical stress on the muscles (Loenneke et al., 2014). This stress mimics the effects of HLRT and promotes an increase in muscle strength. Another way LL-BFRT causes increased muscle strength is likely due to the recruitment of more muscle fibers. Previous research has generally assumed that LL-BFRT increases muscle strength by mobilizing type II fibers (Abe et al., 1985; Cumming et al., 2014). Takashi Abe et al. reported that after LL-BFRT, CSA of type II fiber increased by 27.6% and type I fiber increased by only 5.9% (Abe et al., 1985). KT Cumming et al. found that a significant reduction in type II fiber-associated metabolites after LL-BFRT also confirmed that type II fibers were recruited during training (Cumming et al., 2014). However, this view has been challenged recently. One study found that only type I muscle fibers increased after LL-BFRT was administered to weightlifters (Bjørnsen et al., 2019). Another study measured heat shock proteins in 13 subjects after blood flow restriction training and found that LL-BFRT preferentially stressed type I muscle fibers (Bjørnsen et al., 1985). Therefore, there is currently debate about the type of muscle fibers preferentially affected by LL-BFRT, and invasive biopsy techniques may resolve this contradiction in the future (Davids et al., 2023). Based on our subgroup analysis results, the improvement of upper extremity muscle strength by LL-BFRT was not affected by age compared with LLRT.

Studies have shown that metabolic stress plays an indispensable role in the promotion of LL-BFR to muscle hypertrophy. Growth hormone (GH) is a hormone secreted by the pituitary gland to promote the normal development of the body (Sharifi et al., 2020). It can increase the synthetic and metabolic capacity of muscles by producing insulin-like growth factor (IGF) (Kim et al., 2005). Fry et al. (1985) found that the GH of subjects in the LL-BFRT group was 9 times higher than that in the control group. A study by Takarada et al. (1985) showed that the level of GH after blood flow restriction was 290 times that of people without blood flow restriction. On the other hand, lactate buildup due to blood flow restriction activates mammalian target of rapamycin (mTOR) signaling pathways. mTOR pathway is a key signaling pathway regulating muscle protein synthesis, which can promote muscle protein synthesis and muscle fiber growth (Pearson and Hussain, 2015). After using drugs to inhibit mTOR, (Gundermann et al., 2014) found that LL-BFRT had resistance to muscle protein synthesis, which proved the necessity of mTOR causing muscle hypertrophy in LL-BFRT. In addition, satellite cell (SC) activation is also one of the ways that LL-BFRT causes muscle hypertrophy. Although the training load is small, studies have shown that BFR still causes stress in satellite cells (Nielsen et al., 2017). SCs are activated to promote their proliferation and differentiation and increase the number and size of muscle fibers to achieve muscle proliferation and regeneration (Roberts et al., 2023; Xie et al., 2023). Subgroup analysis found a significant increase in muscle strength but not muscle hypertrophy for LL-BFRT compared to LLRT in young adults. Although they are correlated to a certain extent, increases in muscle strength are not always reflected in increases in muscle size. At the same time, the effect of LL-BFRT on muscle hypertrophy differs across regions compared to LLRT, which also suggests that we should conduct more research on different ethnic groups in this field to explore reasonable training programs.

## 5 Limitations

There are some limitations to this systematic review and meta-analysis. The number of articles on the use of LL-BFRT in upper limb muscles was insufficient to allow for a thorough subgroup analysis in this review. There was wide heterogeneity in cuff pressure, exercise mode, intervention time, and age and gender of participants in the training scheme used in the included research. Measures of muscle strength and hypertrophy also varied from study to study. In the future, uniform research reporting protocols should be applied in the field of blood flow restriction so that the conclusions can be applied into clinical practice. The sample size of the selected articles is generally not high, so the small sample size effect is likely to appear in the conclusion. Because the intervention includes exercise, it is difficult to blind the implementer and the subject. Therefore, more clinical trials of high methodological quality and large sample size are needed to verify the conclusions obtained.

## 6 Conclusion

Based on our findings, Low and moderate certainty evidence suggests that LL-BFRT and HLRT have the same effect on upper extremity muscle strength and hypertrophy. Low certainty evidence suggests that LL-BFRT has a significant advantage over LLRT in improving upper extremity muscle strength and hypertrophy. Through further analysis, we found that although the number of studies included was insufficient, the improvement effect of LL-BFRT on upper extremity muscle is influenced by age and region. In general, it is necessary to consider the above two factors when formulating the LL-BFRT training program. The results of this systematic review and meta-analysis indicate that LL-BFRT can be used as an alternative training method to HLRT to increase upper extremity muscle strength and hypertrophy.

## Data Availability

The original contributions presented in the study are included in the article/Supplementary Material, further inquiries can be directed to the corresponding author.
