# Supplementary material for: Effects of upper extremity blood flow restriction training on muscle strength and hypertrophy: a systematic review and meta-analysis
Source: Front Physiol. 2025 Jan 6;15:1488305. doi: 10.3389/fphys.2024.1488305 (PMC11743574; doi:10.3389/fphys.2024.1488305)
Supplement: Supplementary file 1 [file Table1.docx]

**Supplementary table 1:** Search strategy and results in databases

| **Database** | **Search strategy** | **Results** |
| --- | --- | --- |
| Pubmed | ("Blood Flow Restriction Therapy"[MeSH Terms] OR ("Blood Flow Restriction Therapy"[MeSH Terms] OR ("blood"[All Fields] AND "flow"[All Fields] AND "restriction"[All Fields] AND "therapy"[All Fields]) OR "Blood Flow Restriction Therapy"[All Fields] OR ("bfr"[All Fields] AND "therapy"[All Fields]) OR "bfr therapy"[All Fields] OR ("Blood Flow Restriction Therapy"[MeSH Terms] OR ("blood"[All Fields] AND "flow"[All Fields] AND "restriction"[All Fields] AND "therapy"[All Fields]) OR "Blood Flow Restriction Therapy"[All Fields] OR ("bfr"[All Fields] AND "therapies"[All Fields])) OR ("Blood Flow Restriction Therapy"[MeSH Terms] OR ("blood"[All Fields] AND "flow"[All Fields] AND "restriction"[All Fields] AND "therapy"[All Fields]) OR "Blood Flow Restriction Therapy"[All Fields] OR ("therapy"[All Fields] AND "bfr"[All Fields]) OR "therapy bfr"[All Fields]) OR ("Blood Flow Restriction Therapy"[MeSH Terms] OR ("blood"[All Fields] AND "flow"[All Fields] AND "restriction"[All Fields] AND "therapy"[All Fields]) OR "Blood Flow Restriction Therapy"[All Fields] OR ("blood"[All Fields] AND "flow"[All Fields] AND "restriction"[All Fields] AND "training"[All Fields]) OR "blood flow restriction training"[All Fields]) OR ("Blood Flow Restriction Therapy"[MeSH Terms] OR ("blood"[All Fields] AND "flow"[All Fields] AND "restriction"[All Fields] AND "therapy"[All Fields]) OR "Blood Flow Restriction Therapy"[All Fields] OR ("blood"[All Fields] AND "flow"[All Fields] AND "restriction"[All Fields] AND "exercise"[All Fields]) OR "blood flow restriction exercise"[All Fields]))) AND ("Upper Extremity"[MeSH Terms] OR ("Upper Extremity"[MeSH Terms] OR ("upper"[All Fields] AND "extremity"[All Fields]) OR "Upper Extremity"[All Fields] OR ("extremities"[All Fields] AND "upper"[All Fields]) OR "extremities upper"[All Fields] OR ("Upper Extremity"[MeSH Terms] OR ("upper"[All Fields] AND "extremity"[All Fields]) OR "Upper Extremity"[All Fields] OR ("upper"[All Fields] AND "extremities"[All Fields]) OR "upper extremities"[All Fields]) OR ("Upper Extremity"[MeSH Terms] OR ("upper"[All Fields] AND "extremity"[All Fields]) OR "Upper Extremity"[All Fields] OR ("membrum"[All Fields] AND "superius"[All Fields])) OR ("Upper Extremity"[MeSH Terms] OR ("upper"[All Fields] AND "extremity"[All Fields]) OR "Upper Extremity"[All Fields] OR ("upper"[All Fields] AND "limb"[All Fields]) OR "upper limb"[All Fields]) OR ("Upper Extremity"[MeSH Terms] OR ("upper"[All Fields] AND "extremity"[All Fields]) OR "Upper Extremity"[All Fields] OR ("limb"[All Fields] AND "upper"[All Fields]) OR "limb upper"[All Fields]) OR ("Upper Extremity"[MeSH Terms] OR ("upper"[All Fields] AND "extremity"[All Fields]) OR "Upper Extremity"[All Fields] OR ("limbs"[All Fields] AND "upper"[All Fields]) OR "limbs upper"[All Fields]) OR ("Upper Extremity"[MeSH Terms] OR ("upper"[All Fields] AND "extremity"[All Fields]) OR "Upper Extremity"[All Fields] OR ("upper"[All Fields] AND "limbs"[All Fields]) OR "upper limbs"[All Fields]) OR ("Upper Extremity"[MeSH Terms] OR ("upper"[All Fields] AND "extremity"[All Fields]) OR "Upper Extremity"[All Fields] OR ("extremity"[All Fields] AND "upper"[All Fields]) OR "extremity upper"[All Fields]))) | 148 |
|  | ("Blood Flow Restriction Therapy"[MeSH Terms] OR ("Blood Flow Restriction Therapy"[MeSH Terms] OR ("blood"[All Fields] AND "flow"[All Fields] AND "restriction"[All Fields] AND "therapy"[All Fields]) OR "Blood Flow Restriction Therapy"[All Fields] OR ("bfr"[All Fields] AND "therapy"[All Fields]) OR "bfr therapy"[All Fields] OR ("Blood Flow Restriction Therapy"[MeSH Terms] OR ("blood"[All Fields] AND "flow"[All Fields] AND "restriction"[All Fields] AND "therapy"[All Fields]) OR "Blood Flow Restriction Therapy"[All Fields] OR ("bfr"[All Fields] AND "therapies"[All Fields])) OR ("Blood Flow Restriction Therapy"[MeSH Terms] OR ("blood"[All Fields] AND "flow"[All Fields] AND "restriction"[All Fields] AND "therapy"[All Fields]) OR "Blood Flow Restriction Therapy"[All Fields] OR ("therapy"[All Fields] AND "bfr"[All Fields]) OR "therapy bfr"[All Fields]) OR ("Blood Flow Restriction Therapy"[MeSH Terms] OR ("blood"[All Fields] AND "flow"[All Fields] AND "restriction"[All Fields] AND "therapy"[All Fields]) OR "Blood Flow Restriction Therapy"[All Fields] OR ("blood"[All Fields] AND "flow"[All Fields] AND "restriction"[All Fields] AND "training"[All Fields]) OR "blood flow restriction training"[All Fields]) OR ("Blood Flow Restriction Therapy"[MeSH Terms] OR ("blood"[All Fields] AND "flow"[All Fields] AND "restriction"[All Fields] AND "therapy"[All Fields]) OR "Blood Flow Restriction Therapy"[All Fields] OR ("blood"[All Fields] AND "flow"[All Fields] AND "restriction"[All Fields] AND "exercise"[All Fields]) OR "blood flow restriction exercise"[All Fields]))) AND ("Arm"[MeSH Terms] OR ("Arm"[MeSH Terms] OR "Arm"[All Fields] OR "arms"[All Fields]) OR ("Arm"[MeSH Terms] OR "Arm"[All Fields] OR ("upper"[All Fields] AND "Arm"[All Fields]) OR "upper arm"[All Fields]) OR ("Arm"[MeSH Terms] OR "Arm"[All Fields] OR ("Arm"[All Fields] AND "upper"[All Fields]) OR "arm upper"[All Fields]) OR ("Arm"[MeSH Terms] OR "Arm"[All Fields] OR ("arms"[All Fields] AND "upper"[All Fields]) OR "arms upper"[All Fields]) OR ("Arm"[MeSH Terms] OR "Arm"[All Fields] OR ("upper"[All Fields] AND "arms"[All Fields]) OR "upper arms"[All Fields]) OR ("Arm"[MeSH Terms] OR "Arm"[All Fields] OR "brachium"[All Fields]) OR ("Arm"[MeSH Terms] OR "Arm"[All Fields] OR "brachiums"[All Fields]) OR "Axilla"[MeSH Terms] OR "Elbow"[MeSH Terms] OR "Shoulder"[MeSH Terms]) | 153 |
| Web of Science | Query#1  (Topic)orBFR Therapy(Topic)orBFR Therapies(Topic)orTherapy, BFR(Topic)orBlood Flow Restriction Training(Topic)orBlood Flow Restriction Exercise(Topic)andPreprint Citation Index(Exclude – Database)  Query#2  Upper Extremity(Topic)orExtremities, Upper(Topic)orUpper Extremities(Topic)orMembrum superius(Topic)orUpper Limb(Topic)orLimb, Upper(Topic)orLimbs, Upper(Topic)orUpper Limbs(Topic)orExtremity, Upper(Topic)andPreprint Citation Index(Exclude – Database)  #1 AND #2 | 147 |
|  | Query#1  Blood Flow Restriction Therapy(Topic)orBFR Therapy(Topic)orBFR Therapies(Topic)orTherapy, BFR(Topic)orBlood Flow Restriction Training(Topic)orBlood Flow Restriction Exercise(Topic)andPreprint Citation Index(Exclude – Database)  Query#2  Arm(Topic)orArms(Topic)orUpper Arm(Topic)orArm, Upper(Topic)orUpper Arms(Topic)orBrachium(Topic)orArms, Upper(Topic)orBrachiums(Topic)orAxilla(Topic)orElbow(Topic)orShoulder(Topic)andPreprint Citation Index(Exclude – Database)  #1 AND #2 | 314 |
| The cochrane Library | #1 MeSH descriptor: [Blood Flow Restriction Therapy] explode all trees  #2 Blood Flow Restriction Training  #3 Blood Flow Restriction Exercise  #4 BFR Therapy  #5 Therapy, BFR  #6 BFR Therapies  #7 #1 or #2 or #3 or #4 or #5 or #6  #8 MeSH descriptor: [Upper Extremity] explode all trees  #9 Extremity, Upper; Upper Limbs  #10 Membrum superius  #11 Upper Limb  #12 Upper Extremities  #13 Extremities, Upper  #14 Limbs, Upper  #15 Limb, Upper  #16 #8 or #9 or #10 or #11 or #12 or #13 or #14 or #15  #17 #7 and #16 | 213 |
|  | #1 MeSH descriptor: [Blood Flow Restriction Therapy] explode all trees  #2 Blood Flow Restriction Training  #3 Blood Flow Restriction Exercise  #4 BFR Therapy  #5 Therapy, BFR  #6 BFR Therapies  #7 #1 or #2 or #3 or #4 or #5 or #6  #8 MeSH descriptor: [Arm] explode all trees  #9 Brachium  #10 Arms, Upper  #11 Arm, Upper  #12 Arms  #13 Upper Arm  #14 Brachiums  #15 Upper Arms  #16 MeSH descriptor: [Shoulder] explode all trees  #17 Shoulders  #18 MeSH descriptor: [Elbow] explode all trees  #19 #8 or #9 or #10 or #11 or #12 or #13 or #14 or #15 or #16 or #17 or #18  #20 #7 and #19 | 602 |
| Embase | #1. 'blood flow restriction therapy'/exp OR 'blood flow restriction therapy' OR 'bfr therapy'/exp OR 'bfr therapy' OR 'bfr therapies' OR 'therapy, bfr' OR 'blood flow restriction training'/exp OR 'blood flow restriction training' OR 'blood flow restriction exercise'/exp OR 'blood flow restriction exercise'  #2. 'upper extremity'/exp OR 'upper extremity' OR 'extremities, upper' OR 'upper extremities' OR 'membrum superius' OR 'upper limb'/exp OR 'upper limb' OR 'limb, upper' OR 'limbs, upper' OR 'upper limbs' OR 'extremity, upper'  #3. #1 AND #2 | 55 |
|  | #1. 'blood flow restriction therapy'/exp OR 'blood flow restriction therapy' OR 'bfr therapy'/exp OR 'bfr therapy' OR 'bfr therapies' OR 'therapy, bfr' OR 'blood flow restriction training'/exp OR 'blood flow restriction training' OR 'blood flow restriction exercise'/exp OR 'blood flow restriction exercise'  #2. 'arm'/exp OR 'arm' OR 'arms'/exp OR 'arms' OR 'upper arm'/exp OR 'upper arm' OR 'arm, upper' OR 'arms, upper' OR 'upper arms' OR 'brachium' OR 'brachiums' OR 'shoulder'/exp OR 'shoulder' OR 'elbow'/exp OR 'elbow'  #3. #1 AND #2 | 72 |

**Supplementary table 2:** Measurement method of the outcome in muscle strength and hypertrophy

| **Study** | **Outcome** | **Measurement method** |
| --- | --- | --- |
| Thiago Cândido Alves et al.,2021 | 1RM of elbow flexion and extension | The process of 1RM test is for the individual to perform repetitions until failure, without exceeding 10 repetitions. The subjects had up to three attempts at three-minute intervals to perform the test within the established repetition limit. |
| Christopher R. Brandner et al.,2019 | 1RM of elbow flexion | Participants completed all 1RM tests through a full range of motion. Before each 1RM test, participants warmed up at 50% of their estimated 1RM. Single repetition lifts were conducted with progressively heavier loads until failure, defined as the final load that could be successful lifted with correct technique where an additional 0.5 - 5.0 kg could not be successfully lifted. Rest intervals between 1RM attempts were dependent on participant readiness but ranged from 2 - 5 min, while not more than four to six attempts were completed during any test. |
|  | MTH of biceps and triceps brachii | B-mode ultrasonographic evaluation of skeletal MTH. MTH was determined as the distance between the adipose-muscle interface and muscle-bone interface from the ultrasound image in accordance as per previous protocols. Briefly, the anatomical landmarks of the sites were as follows: on the anterior and posterior surface equal to 60% distal between the lateral epicondyle of the humerus and the acromial process of the scapula; Pectoralis major: at the clavicular midpoint and between the third and fourth costa; |
| Christian Linero et al.,2021 | 1RM of elbow flexion and extension | 1RM was measured to determine change in muscle strength based on ACSM procedure. For 1RM biceps curl test, unilateral dumbbell biceps curl with dominant arm were tested, and for 1RM triceps extension, authors used cable cross-over machine. |
| Tomohiro Yasuda et al.,2015(1) | MVIC of elbow flexion and extension | MVIC of the elbow flexors and elbow extension was measured twice by a dynamometer. |
|  | CSA of biceps and triceps brachii | CSA was obtained using an MRI scanner. Skeletal muscle tissue CSA was measured for elbow flexors at 6cm above the elbow joint and for elbow extensors at 16cm above the elbow joint. |
| Ethan C. Hill et al.,2020 | MVIC of elbow flexion | The participants performed a warm-up consisting of 10 submaximal, reciprocal concentric forearm flexion - extension muscle actions at 120°s^−1^. Following warm-up, the participants rested for 5 min and then performed two randomly ordered maximal concentric and isometric muscle actions of the forearm flexors and extensors at 120°s^−1^ to determine the concentric peak torque and MVIC values, respectively. The highest concentric peak torque and MVIC torque produced during each of the two attempts was used for further analyses. The concentric muscle actions were performed through a 120° range of motion and the MVIC muscle actions were performed at 45° of flexion at the elbow sustained for a period of 3 s. |
|  | CSA and MTH of biceps brachii | CSA and MTH were assessed via ultrasound prior to each testing visit. Ultrasound images of the trained arm were obtained using a portable brightness mode (B-mode) ultrasound-imaging device a multi-frequency linear-array probe. All ultrasound measurements were  performed at a sampling rate of 10 MHz and at a gain of 58 dB. |
| Yanhong Su et al.,2024 | PT of elbow flexion | PT of elbow flexion was measured by isometric muscle strength test. Isometric elbow test for 5 s, with the elbow joint set to a flexion angle of 90° |
| Elizabeth WELLS et al.,2019 | 1RM of elbow flexion | The participant chose a weight in which they could not complete more than nine consecutive repetitions. If the participant completed nine or more repetitions, they rested for two minutes and completed the task again with an increased weight by 2.5-5 kg. |
|  | CSA and MTH of biceps brachii | MTH and CSA of the biceps brachii was measured using an ultrasound unit. |
| Ryan P. Lowery et al.,2014 | MTH of biceps brachii | Muscle thickness of each participant on their dominant arm was determined via the ultrasound by measuring the total distance of the long and short head of the biceps brachii as well as the fascia between the two muscles located midway between the head of the humerus and lateral epicondyle. |
| Tomohiro Yasuda et al.,2011 | MVIC of elbow extension | MIVC of the elbow extension was determined by using an isokinetic dynamometer. |
|  | CSA of triceps brachii | MRI images were prepared using a 0.2-T scanner. Participants rested quietly in the magnet bore in a supine position, with their arms extended along their trunk. Continuous transverse images with 10-mm slice thickness were obtained from both upper arms of the body. Triceps brachii muscle CSAs of three contiguous slices for muscle belly were averaged for statistical analysis. |
| Dilara Kara et al.,2024 | MTH of biceps brachii | MVIC was assessed at 60 degrees/s and 180 degrees/s using isokinetic dynamometer. |
| Tomohiro Yasuda et al.,2015(2) | MVIC of elbow flexion and extension | MVIC of the elbow flexors and extensors were measured by a dynamometer. |
|  | CSA of biceps and triceps brachii | CSA was obtained using an MRI scanner. A T-1 weighted, spin-echo, axial plane sequence was performed with a 500-ms repetition time and a 23-ms echo time. Participants rested quietly in the magnet bore in a supine position, with their arms extended along their trunk. Continuous transverse images with 10-mm slice thickness were obtained from both upper arms of the body. Muscle CSA was measured for elbow flexion and extension at 6cm and 16cm above the elbow joint |

Abbreviations: 1RM, one repetition maximum; MTH, muscle thickness; MVIC, maximum voluntary isomeric contraction; CSA, cross-sectional area; PT, peak torque, MRI, magnetic resonance imaging.
